# Development of Waste Polystyrene-Based Copper Oxide/Reduced Graphene Oxide Composites and Their Mechanical, Electrical and Thermal Properties

**DOI:** 10.3390/nano11092372

**Published:** 2021-09-13

**Authors:** Waqas Ahmad, Qaizar Ahmad, Muhammad Yaseen, Imtiaz Ahmad, Fida Hussain, Badrul Mohamed Jan, Rabia Ikram, Minas M. Stylianakis, George Kenanakis

**Affiliations:** 1 Institute of Chemical Sciences, University of Peshawar, Peshawar 25120, Pakistan; qaizarmwt92@gmail.com (Q.A.); myyousafzai@gmail.com (M.Y.); patwar2001@yahoo.co.in (I.A.); 2 Department of Chemical and Energy Engineering, Pak-Austria Fachhochschule, Institute of Applied Science & Technology, Haripur 22621, Pakistan; fida.hussain@fcm3.paf-iast.edu.pk; 3 Department of Chemical Engineering, University of Malaya, Kuala Lumpur 50603, Malaysia; 4 Institute of Electronic Structure and Laser, Foundation for Research and Technology-Hellas, N. Plastira 100, Vasilika Vouton, GR-700 13 Heraklion, Greece; stylianakis@iesl.forth.gr (M.M.S.); gkenanak@iesl.forth.gr (G.K.)

**Keywords:** reduced graphene oxide, copper oxide, waste polystyrene matrix, electrical conductance

## Abstract

The current study reports the effect of different wt. ratios of copper oxide nanoparticle (CuO-NPs) and reduced graphene oxide (rGO) as fillers on mechanical, electrical, and thermal properties of waste polystyrene (WPS) matrix. Firstly, thin sheets of WPS-rGO-CuO composites were prepared through solution casting method with different ratios, i.e., 2, 8, 10, 15 and 20 wt.% of CuO-NPs and rGO in WPS matrix. The synthesized composite sheets were characterized by Fourier transform infrared spectroscopy (FTIR), energy dispersive X-ray (EDX), X-ray diffraction (XRD) analysis, scanning electron microscopy (SEM) and thermal gravimetric analysis (TGA). The electrical conductance and mechanical strength of the prepared composites were determined by using LCR meter and universal testing machine (UTM). These properties were dependent on the concentrations of CuO-NPs and rGO. Results display that the addition of both fillers, i.e., rGO and CuO-NPs, collectively led to remarkable increase in the mechanical properties of the composite. The incorporation of rGO-CuO: 15% WPS sample, i.e., WPS-rGO-CuO: 15%, has shown high mechanical strength with tensile strength of 25.282 MPa and Young modulus of 1951.0 MPa, respectively. Similarly, the electrical conductance of the same composite is also enhanced from 6.7 × 10^−14^ to 4 × 10^−7^ S/m in contrast to WPS at 2.0 × 10^6^ Hz. The fabricated composites exhibited high thermal stability through TGA analysis in terms of 3.52% and 6.055% wt. loss at 250 °C as compared to WPS.

## 1. Introduction

Over the years, extensive research has been carried out on the polymeric composites to improve their electrical, thermal and mechanical properties [1]. Polymers are profoundly demanded materials, having simple structure, wide accessibility and produced from low-cost materials [2,3]. Due to their extraordinary properties, such as good insulation, high mechanical properties with light mass, climate impediment, transport properties and ease of fabrication into variety of shapes and forms, polymers reveal a wide variety of applications [3,4]. However, some of the polymer properties are undesirable, such as poor thermal and oxidation stability, low mechanical strength and poor electrical conductance, which restricts their usage in certain fields [5,6]. Therefore, material researchers are endeavoring to upgrade the properties of polymers for desirable applications.

In recent years, polymer composites were introduced by consolidating different sorts of inorganic fillers into polymeric frameworks. They revealed some alluring properties which make them suitable for many advanced applications, such as aviation, the automotive industry and sensors or microelectronic industries [7,8]. Among various fillers, different ceramic materials have exceptional characteristic combinations, including high erosion obstruction, outrageous temperature stability, high and flexible modulus and efficient electrical and thermal conductivity, which sufficiently improve the second-rate properties of polymeric materials [9,10,11]. Similarly, rGO is also used as a filler in polymer frameworks due to its high explicit surface area, restored capacitance and interesting electron transport properties [12]. 

Studies have shown enhanced thermal and electrical properties of various types of polymeric composites doped with graphene oxide (GO) [13]. Nano-sized CuO, is an attractive material which finds wide applications in many fields on account of its attractive properties such as its nano size range, specific surface area and outstanding electrical, thermal and mechanical properties [14]. It is a p-type semiconductor, having promising electrical, thermal and magnetic properties. It has been extensively used to fabricate polymer nanocomposites for numerous applications such as catalysis, solar energy conversion, gas sensors, supercapacitors and semiconductors [15,16,17]. For example, polyvinyl alcohol (PVA)-doped CuO nanocomposite was formed by solution casting method in which PVA was added as a semi crystalline hydrophilic polymer matrix and CuO-NPs was added as a filler. The resulting nanocomposite was comprised of 2.0 wt.% CuO-NPs which showed excellent results of alternating current (AC) conductivity at varying frequency [18]. 

PS is a synthetic polymer composed of an aromatic hydrocarbon monomer called styrene. It is a hard, rigid, transparent and inexpensive thermoplastic [19]. PS has been widely used for making disposable cutlery, food trays, drinking cups, plastic models, egg boxes, audio/video cassettes, toys, general household appliances, light diffusers, electronics and a wide range household products [20]. The principle limitations of PS due to its brittleness, poor electrical conductivity, low chemical resistance and mechanical strength [21], which decrease its application in several fields. Literature studies exhibit that such substandard characteristics of polymers can be upgraded by incorporating a variety of fillers into the polymer matrix [22]. Electrical conductivity is a highly desirable property of polymeric composites, based on which they are incorporated into gas sensing materials [23], electronic devices [24], energy storage [25], electrochemical tools [26], photovoltaic cells [27], fuel cells [23], transistors [24], electromagnetic shielding films [28] and optoelectronic devices [29]. 

Due to the large-scale production and usage of PS, the vast majority is dumped into the environment as waste, which causes serious environmental concerns. Owing to its non-biodegradable nature, this growing solid waste is currently handled in different ways, such as landfills and incineration, however, these procedures, in turn, generate more serious pollution. In order to reutilize the WPS in a productive way, it can be recycled into value-added materials with improved properties for diverse applications. 

In this study, expanded WPS was collected from street wastes. It was transformed into a composite material by fortification with various amounts of rGO and CuO-NPs as fillers. To summarize, we utilized this waste to produce a novel material, in order to minimize its environmental impacts. The combination of rGO and CuO-NPs on PS matrix was identified by examining different characteristics of fabricated composites including mechanical strength, thermal stability, composition and morphology. The composite and precursors were characterized by FTIR, XRD, EDX and SEM analysis. In addition, electrical properties of the composites were also investigated. 

## 2. Experimental

### 2.1. Chemical Reagent

All the chemicals and reagents used were of analytical grade. Graphite powder, potassium permanganate (KMnO_4_), sulfuric acid (H_2_SO_4_) 98%, hydrogen peroxide (H_2_O_2_) 30%, ammonia (NH_4_OH) 25–28%, L-ascorbic acid (vitamin c), sodium hydroxide (NaOH), copper nitrate (Cu(NO_3_)_2_, potassium hydroxide (KOH), and chloroform were purchased from Sigma-Aldrich, St. Louis, MO, USA. Waste Polystyrene (PS) was collected (disposable Styrofoam articles) from street wastes in University Campus of Peshawar. 

### 2.2. Synthesis of Copper Oxide Nanoparticles

CuO-NPs were prepared through co-precipitation method, where copper nitrate (Cu(NO_3_)_2_) was used as a metal precursor and NaOH as reducing agent. For the synthesis of CuO-NPs, 100 mL of (Cu(NO_3_)_2_) (0.1 M) solution was placed into a conical flask and NaOH (0.1 M) was added dropwise under vigorous stirring until pH of the solution reached to 14. The suspension was recovered through filtration and washed several times with deionized water and methanol until pH reached 7. The prepared particles were dried and then calcined at 500 °C for 4 h [30].

### 2.3. Synthesis of GO

GO was synthesized using modified Hummer method from fine graphite powder as reported in the literature [30,31]. Sodium nitrate (2 g) was gradually added to 4 g of graphite and 92 mL of H_2_SO_4_ was added under vigorous stirring. The mixture was placed into the ice bath to lower the temperature of the prepared mixture to below 10 °C. About 23 g potassium permanganate (KMnO_4_) was added to the mixture and stirred for 30 min. The suspension was allowed to settle overnight. Further, 184 mL of deionized water was added to the suspension and heated up to 95 °C for 15 min, and 30% hydrogen peroxide (5–20 mL) was added dropwise till the color of the paste turned yellow, producing GO [31]. The GO was dispersed in 500 mL deionized water and sonicated along with 10 g/L ascorbic acid (vitamin C) as a reducing agent for 1 h. The suspension was heated at temperature of 95 °C for 1 h. The brown color of GO subsequently changed to dark precipitate of rGO. The precipitates were separated by vacuum filtration, washed with deionized water and then dried in the oven overnight [32].

### 2.4. Synthesis of WPS-rGO-CuO Composites

The WPS-rGO-CuO composite material was synthesized by incorporating different amounts of the rGO and CuO-NPs as filler materials into the WPS polymer matrix. The WPS was dissolved in the chloroform and calculated amounts of rGO and CuO required for 2, 8, 10, 15 and 20 wt.% loading were added into the solution separately. The fillers were dispersed in the WPS matrix solution through sonication for 5 h, until a homogenized suspension was obtained. The homogeneous mixture was poured into glass Petri dishes to draw sheets of the composite material after drying. The fabricated composite samples prepared were coded as: WPS-rGO-CuO: 2%, WPS-rGO-CuO: 8%, WPS-rGO-CuO: 10%, WPS-rGO-CuO: 15% and WPS-rGO-CuO: 20%.

### 2.5. Structural Characterization of Composite 

The prepared composites were characterized by SEM, EDX, FTIR and XRD analysis. SEM-EDX were carried out using a scanning electron microscope (Model JEOL-Jsm-5910; Tokyo, Japan), while XRD characterizations were conducted using X-ray diffractometer (XRD; model JDX-9C, JOEL, Tokyo, Japan) with CuKα radiation (1.54178 A° wavelength) and a nickel filter. FTIR analysis of the catalyst was carried out by FTIR spectrophotometer (Schimadzu-A60, Tokyo, Japan).

### 2.6. Mechanical and Thermal Properties

Mechanical properties including tensile strength, Young modulus, rigidity, modulus of elasticity, elongation and hardness of the WPS and its composites were determined through universal testing machine (UTM). The composite samples of definite dimensions (20.0 × 9.0 × 0.13 mm) were used for the analysis. A steady stress was applied on the sample, and the level of strain was recoded till the sample fractured. Different mechanical parameters such as tensile strength, % elongation, and Young modulus were calculated from stress strain relationship.

The thermal stability of WPS, rGO, CuO-NPs and their composites was evaluated by TGA analyzer (Perkin Elmer). The TGA investigation was carried out in the temperature range of 30–600 °C at a warming pace of 10 °C per min.

### 2.7. Electrical Conductance

The AC electrical conductance of the composite samples was investigated by an LCR meter under various frequencies ranging from 25 to 1 MHz and different temperatures. Among the sheets of different WPS-rGO-CuO composite samples, each having 5.5 mm thickness were coated with silver in order to make good contact and were mounted between two copper electrodes in a special sample holder. The sample holder was inserted into a small furnace and AC conductivity was measured by LCR meter. The measurement was carried out in the frequency range of 25 to 1 MHz under a controlled temperature between 303 and 348 K. The AC electrical conductance was plotted verses increasing order of frequency.

## 3. Results and Discussion

### 3.1. Characterization of Composite

**Figure 1:**
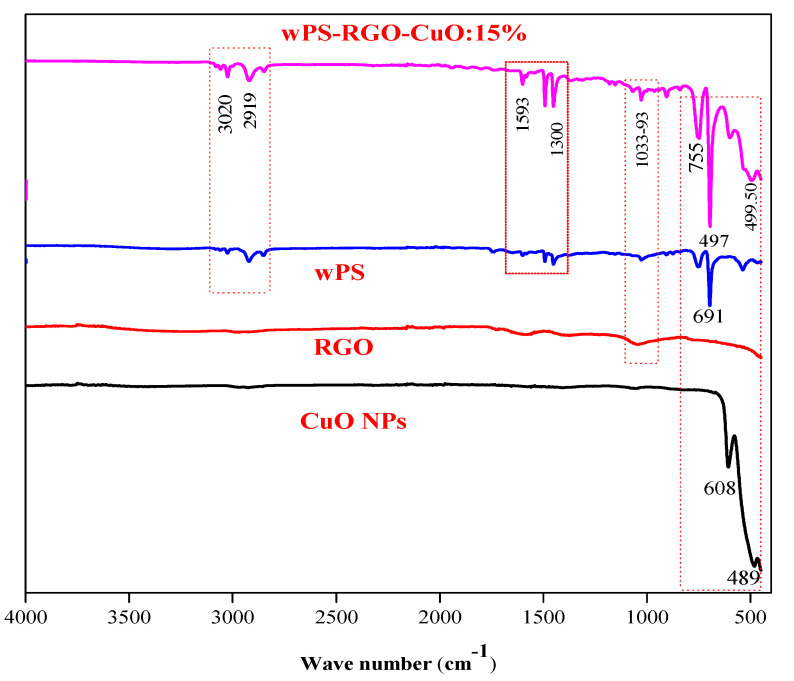
FTIR spectra of CuO-NPs, rGO, WPS and WPS-rGO-CuO: 15% composite.

FTIR spectra of CuO-NPs, rGO, WPS and the fabricated composite samples are shown in Figure 1. The spectra of CuO-NPs indicate peaks at 489 and 608 cm^−1^, which unveils the vibrations of Cu(II)-O bonds, an intense peak at 608 cm^−1^ is characteristic of CuO-NPs [33]. The spectrum of rGO also indicates two peaks at 1018 and 1663 cm^−1^ which can be attributed to the vibrations of C–O and aromatic C=C configurations, respectively [34]. A broad and less intense peak centered at 3448 cm^−1^ corresponds to the O-H stretching vibration [35]. These configurations confirm the successful synthesis of rGO from graphite. In the FTIR spectrum of WPS, two peaks are located at 3020 cm^−1^ and 2919 cm^−1^, corresponding to aromatic C–H stretching vibrations and aliphatic C-H stretching vibrations, respectively. The peaks that appeared at 1300 and 1593 cm^−1^ are assigned to aromatic C–H bond stretching vibrations and aromatic C=C bonds. The intense peaks at 1034 cm^−1^ corresponds C–O configurations which may be due to some oxidized impurities in the used WPS. Peaks centered at 755, 691 and 546 cm^−1^ display aromatic C–H deformation vibrations [36,37,38]. All these configurations are affiliated to the organization of WPS. Major peaks in the spectrum of WPS-rGO-CuO: 15% composite appear positioned at 3012, 2919, 1593, 1320, 776, 699 and 988 cm^−1^, which confirms the configuration of the WPS. The peak at 591.99 cm^−1^ confirms the association of CuO-NPs with the WPS, Similarly, the peaks positioned at around 15,993, 1500 and 1033 cm^−1^ confirm the interaction of rGO with the PS matrix [39]. Thus, a hybrid composite synthesis is confirmed in correspondence with the structure of WPS, rGO and CuO-NPs, respectively.

**Figure 2:**
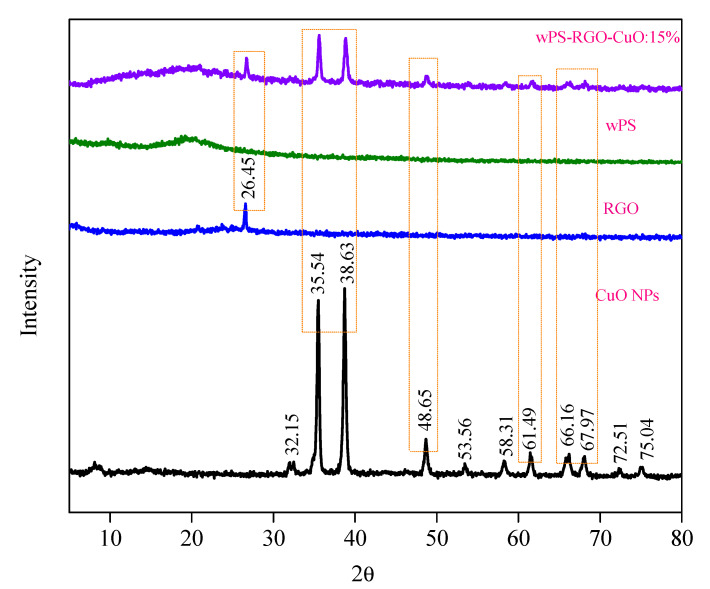
XRD pattern of CuO-NPs, rGO, WPS and WPS-rGO-CuO: 15%.

XRD analysis of fillers, WPS and its composite samples was carried out to study and evaluate their phase structure. The XRD patterns of CuO-NPs, rGO, WPS and WPS-rGO-CuO: 15% composite is exhibited in Figure 2. The XRD pattern of CuO-NPs indicates that the characteristic diffraction peaks observed at 2θ = 32.58°, 35.47°, 38.97° and 48.74° are assigned to (110), (111), (200) and (202) indices. The XRD analysis indicated a high crystallite size of 19 nm, measured by using the Debye–Scherrer equation [33]. The XRD pattern of CuO-NPs illustrates their crystalline nature and closely matches with standard JCPDS card No. 45-0937 of CuO-NPs [40]. Similarly, the XRD pattern of rGO is displayed in Figure 2, which reveals the broad peak observed at 2θ = 25° is assigned to (002), which clearly confirms the reduction of GO to rGO by ascorbic acid [41].

The XRD pattern of WPS presents two broad peaks at 2θ = 12.6° and 2θ = 19.1°, which confirms the amorphous nature of WPS [35,36,42]. The XRD pattern of WPS-rGO-CuO: 15% composite discloses two peaks of WPS at 2θ = 19.1° and 2θ = 12.6°. Similarly, a peak at 25° at 2θ value is found in the composite, which correlates to the peak of rGO. In the same way, peaks at 2θ = 32.58°, 35.47°, 38.97° and 48.74° can also be detected in the composite which confirms that the CuO-NPs are dispersed in the polymer matrix. From these results it can be suggested that the crystallinity of fillers is maintained in the WPS-rGO-CuO: 15% composite material. The amorphous WPS matrix has been reinforced with crystalline fillers, hence, the composite shows the presence of some crystalline phases which is likely to influence the mechanical properties of the composites. 

**Figure 3:**
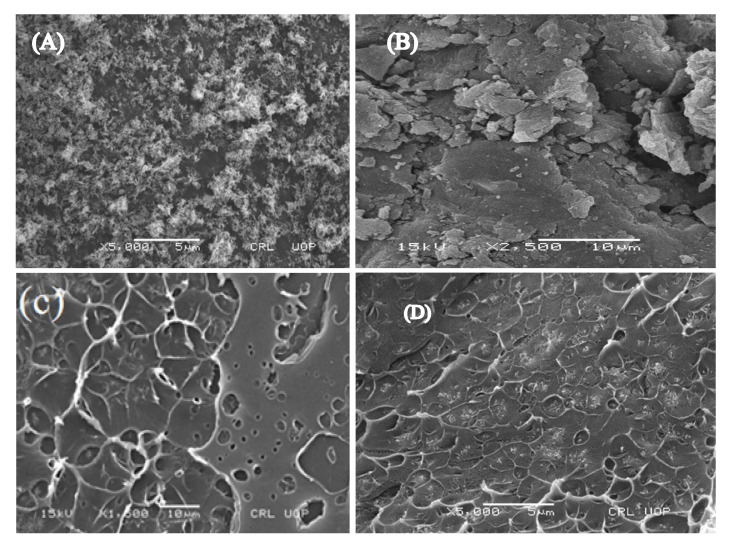
SEM micrographs of (**A**) CuO-NPs, (**B**) rGO, (**C**) WPS, (**D**) WPS-rGO-CuO: 15%.

SEM analysis was carried out to study the morphological nature of the precursors, WPS and its composites. The SEM micrographs of CuO-NPs, rGO, WPS and WPS-rGO-CuO: 15% composite are indicated in Figure 3A–D. The SEM micrograph of CuO-NPs presents nanorod like particles of irregular size. Most of the particles are of small size having length less than 7 nm but some large size particles are also present having size of about 19 nm. It has been found that all particles smaller than 20 nm are desirable for consistent dispersion in the polymer matrix [43]. Similarly, the SEM micrograph of rGO is given in Figure 3B, which reveals that it has thin wrinkles and scaly sheets with porous fluffy network resemblance with spongy like structure. 

The SEM micrograph of WPS displayed in Figure 3C exhibits irregular and rough structure. Deep holes are formed on the surface due to which stripes are separated from each other. WPS was dissolved in toluene and then recycled into sheets; however, when the sample (WPS) is subjected to tear, abrasion and erosion during operation which might have led to changes in the polymer structure, the polymer chains were not successfully twisted together and hence, presents rougher structure rather than a smooth surface.

The SEM pattern of WPS-rGO-CuO: 15% composite in Figure 3D exposes the uniform dispersion of fillers in the matrix. With the incorporation of fillers, deep holes and rough structure of the polymer matrix is modified and the embedded particles in the PS matrix confirm the synthesis of the composite without any agglomerate formation. Conversely, by increasing the concentration of fillers, i.e., 20% into the WPS matrix, agglomerate formation can be seen in the image. Surface structure of the composite with rGO-CuO: 15% in WPS produces high modification and the fillers are more tightly packed with the PS matrix. At this point, the strong association of fillers with matrix is exhibited due to Van der Waals forces and the uniform dispersion, especially in case of WPS-rGO-CuO: 15%, which is due to the vigorous sonication [44]. Since the surface of the composite displays no agglomerates or flakes, this confirms the uniform dispersion of fillers in the matrix; in addition, the texture of the composite seems denser and more compact. 

**Figure 4:**
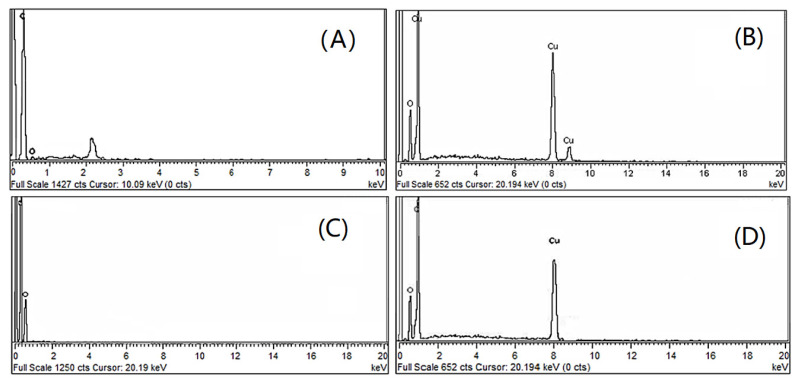
EDX profile of (**A**) WPS, (**B**) CuO-NPs, (**C**) rGO, (**D**) WPS-rGO-CuO: 15%.

The elemental composition of precursors, WPS and its composite samples was carried by EDX analysis. EDX profiles of all samples are given in Figure 4A–D and the elemental composition of the samples is displayed in Table 1. The EDX pattern in Figure 4A shows that the WPS is comprised of 94.30% carbon and 5.70% oxygen. Although hydrogen is also present in WPS, it cannot be detected by EDX analysis. Being organic (hydrocarbon) in nature, the amount of carbon indicated by EDX analysis is in agreement with the theoretical values. Similarly, the EDX analysis of CuO-NPs (Figure 4B) contains 82.64% copper and 17.34% oxygen, which clearly matches with the theoretical amount of copper and oxygen in the CuO-NPs sample [45]. The EDX profile of rGO (Figure 4C) indicates that it carries 91.37% carbon and 8.63% oxygen individually. Likewise, in the case of rGO, the amount of C and O was found to be 91.37 and 8.63%, respectively, which nearly matches with the literature values of carbon and oxygen in rGO samples [46].

EDX profile of WPS-rGO-CuO: 15% displays (Figure 4D) that it is comprised of 89.49% carbon, 5.06% oxygen and 5.44% copper, respectively. Theoretically it has been found that it contains 89.5% carbon, 5.04% oxygen and 5.44% copper. The above theoretically calculated values having close resemblance with experimental values, confirm the synthesis of composite samples.

### 3.2. Mechanical Properties

Mechanical properties including tensile strength, Young modulus, rigidity, modulus of elasticity, elongation and hardness are the physical properties that a material exhibits upon the application of forces. Certain factors, such as composition, component properties, structure and interfacial interactions, greatly affect all these properties. In the current study, mechanical properties of the WPS and its composites were determined through universal testing machine (UTM). The impact of fillers and their proportions in the polymer matrix was studied, for which the sheets of given composite sample having specific dimension were tested by UTM. During mechanical characterization, when stress is applied on the sample, the sample opposes distortion, however, at a certain degree deformation and displacement of the particles begins, which is assigned as a yield point. Further, at a specific increase in pressure permanent deformation happens till the material breaks. The behavior of WPS and the composite material was contemplated, and various parameters were assessed including strain, extension, rigidity and sturdiness. Different mechanical properties of composites containing various amounts of CuO-NPs and rGO in WPS matrix, i.e., 2, 8, 10, 15 and 20% are designated in Table 2. Elongation at break indicates the relationship between increased length and initial length before undergoing cracking pressure, whereas elongation at yields indicates the length at yield point. In Table 2, the data reveals that neat WPS exhibits elongation at break of 3.14 mm, whereas, for the composite containing 2, 8, 10, 15 and 20% CuO-rGO, it had elongation at breaks of 3.23, 1.24, 1.91, 1.16 and 1.18 mm, respectively. Likewise, for WPS, the value of elongation at yield was 0.357 mm, whereas for composites having 2, 8, 10, 15 and 20% of rGO-CuO, the values of elongation at yield were 0.75, 0.43, 0.35, 0.28 and 0.24 mm, respectively. These observations demonstrate that the composite WPS-rGO-CuO: 2% presents higher elongation at break than WPS, which means that the composite WPS-CuO-rGO: 2% is more elastic than WPS. However, WPS-rGO-CuO: 10% entails higher elongation than WPS-rGO-CuO: 8%, whereas, WPS-rGO-CuO: 15% and WPS-rGO-CuO: 20% present lower values of elongation than neat WPS, WPS-rGO-CuO: 2% and WPS-rGO-CuO: 8% composites. It reveals that the composite sample becomes rigid with the addition of 20% CuO and rGO [47]. Elongation to break typically decreases severely with the addition of rigid filler. While elongation of most polymers decreased with the addition of graphene. Similarly, the composites WPS-rGO-CuO: 2% and WPS-rGO-CuO: 8% induce higher elongation at break than WPS while the other three, i.e., WPS-rGO-CuO: 10%, WPS-rGO-CuO: 15% and WPS-rGO-CuO: 20%, showed lower values of elongation at break than WPS.

Similar results are obtained for strain at break and strain at yield, which represents the % increase in length under initial stress before permanent deformation and plastic deformation, respectively. It also exposes the stiffness of the materials. Only in the case of WPS-rGO-CuO: 2%, the percentage elongation is about 37.28% and 80.25% for strain at break and strain at yield, respectively. Whereas, for the remaining composite samples, i.e., PS-rGO-CuO: 8%, WPS-rGO-CuO: 10%, WPS-rGO-CuO: 15% and WPS-rGO-CuO: 20%, the percentage elongation gradually decreases. Likewise, in the case of WPS-rGO-CuO: 2%, the plastic strain at break also shows about 37.50% elongation in length. Due to the addition of rGO-CuO as a filler, in the case of WPS-rGO-CuO: 2%, strain at break and strain at yield both are increased by 37.28 and 28.25, respectively. It represents that the dispersion of filler contents is small enough to make strong interaction with the polymer matrix [48].

Load at break displays the amount of stress or tension which a material can withstand at the time of breaking. Likewise, load at yield indicates the tension a material can withstand before yield point. It can be observed that load at break is higher for the composites than WPS as well as load at yield. The composite WPS-rGO-CuO: 15% produces maximum value of load at break, i.e., 31.78 N, and 63.28 N load at yield for composite WPS-rGO-CuO: 2%. These two ratios, WPS-rGO-CuO: 15% and WPS-rGO-CuO: 2%, entail maximum values, whereas, the remaining ratios present low values and decrease gradually with the addition of CuO and rGO fillers. This shows that WPS-rGO-CuO: 15% composite is tougher than the WPS sample. Additionally, the data also indicate that other composite samples having lower values for load at break are ductile.

**Figure 5:**
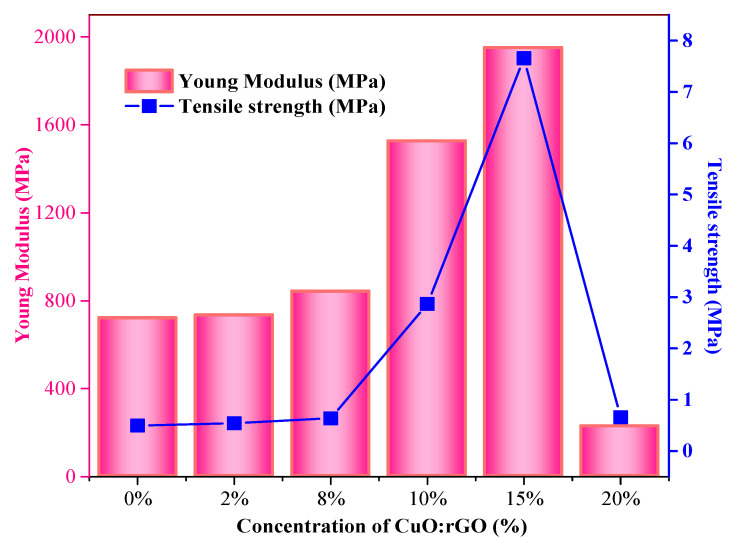
Tensile strength and Young modulus of WPS-rGO-CuO composites with different concentrations of fillers.

Moreover, stress at break represents the ultimate strength of the material, which is the maximum stress a material can withstand before it fractures. Stress at yield, also called yield strength, reveals the maximum stress that materials can withstand before their yield points. The data indicate that the tensile strength of the composite is higher than WPS and gradually increases with the addition of rGO-CuO content up to 7.66 MPa for composite WPS-rGO-CuO: 15%, and then decreases as shown in Figure 5. Whereas, the yield strength of composites also increases linearly by adding rGO-CuO as a filler, and the value of yield strength of all the composite samples, i.e., WPS-rGO-CuO: 2%, WPS-rGO-CuO: 8%, WPS-rGO-CuO: 10%, WPS-rGO-CuO: 15% and WPS-rGO-CuO: 20%, is higher than WPS. Among these ratios, the composite WPS-rGO-CuO: 2% shows a maximum value of 25.28 MPa compared to other composites, because of strong association of the filler material with the PS matrix [49].

Young modulus is one of the most important and fundamental mechanical properties, which is the measure of the resistance to deformation. The data in Table 1 shows that the Young modulus of the composites is higher than the WPS and increases linearly with increasing the loading of fillers. These results confirm that the composite with 15% CuO and rGO fillers shows the maximum value of modulus, i.e., 1951.0 MPa and below 15% loading of fillers. Young modulus increase with GO dispersion is evident for all polymers, but is more pronounced for elastomeric polymers [50]. The value of Young modulus decreases because of weak interaction of filler materials with the WPS matrix.

Literature studies state that key properties such as tensile behavior, % elongation and Young modulus for some other polymer composites were found to be improved with the addition of various types of fillers [40,49]. For example, PS/sisal composites in which sisal fibers are used as a filler and PS as a matrix shows high mechanical properties, such as Young modulus, tensile strength, etc. with the addition of 10% sisal fibers as fillers [51]. Similarly, PS-doped boron nitride nanotube (PS/BNNT) composites also generate modification and enhancement in the key properties with the loading of 1% BNNT. This incorporation of 1 wt.% of BNNT into the PS resulted in elastic modulus increases for the composite material of up to 21% [52]. On the basis of given results in Table 2, the composites WPS-rGO-CuO: 2% and WPS-rGO-CuO: 15% showed enhanced mechanical properties and these two samples were selected for further investigation.

**Figure 6:**
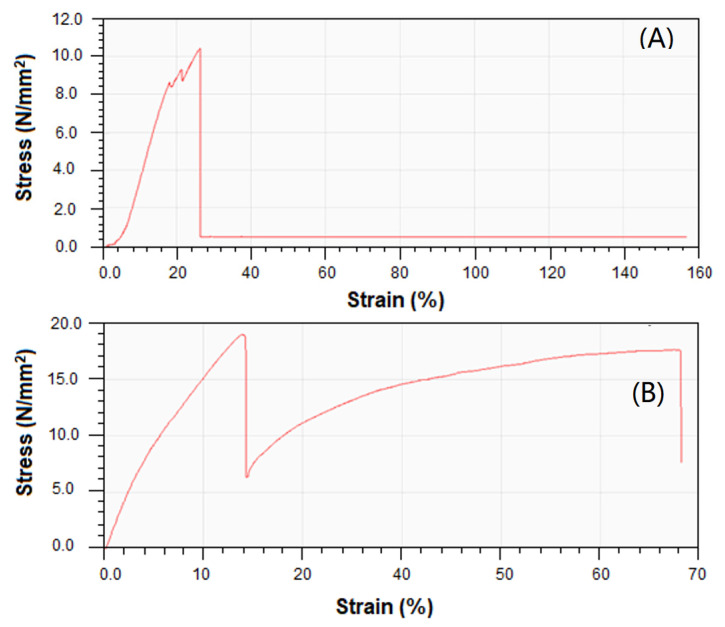
Stress–strain curves of (**A**) WPS and (**B**) WPS-rGO-CuO: 15% composite.

The stress–strain curves of the WPS and WPS-rGO-CuO: 15% composite are displayed in Figure 6, which show that the resistance of composite to the applied stress is markedly increased when compared to that of WPS. Various parameters of mechanical strength based on these curves are already described above. Table 3 summarizes literature studies for the comparative analysis of mechanical properties of WPS-rGO-CuO: 15% composites.

### 3.3. Thermal Properties

**Figure 7:**
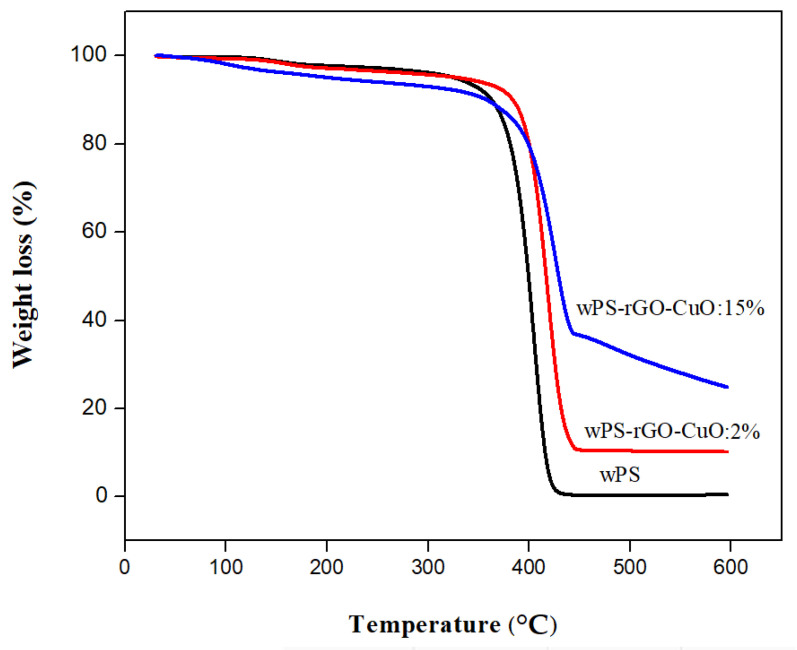
TGA profile of WPS, WPS-rGO-CuO: 2% and WPS-rGO-CuO: 15%.

TGA analysis was performed to study the thermal behavior and stability of WPS and its composites. In Figure 7, the thermograms of WPS and its composites are shown and % wt. loss in composite samples at different temperature are given in Table 4. It can be seen that the thermogram indicates thermal decomposition of all the synthesized samples which was carried out in the temperature range of 50–600 °C [55]. Earlier studies demonstrate that the thermal degradation of the samples was observed at three distinct temperature ranges [56]. The data in Table 4 shows % wt. loss and thermal stability of WPS and its composites in two temperature ranges. The first mass loss (3.52 and 6.07%) occurred at 50–250 °C in terms of WPS-rGO-CuO:2% and WPS-rGO-CuO:15% in contrast to WPS with 8.67% wt. loss, which is due to removal of moisture [57]. The second mass loss is due to pyrolysis and degradation of the polymeric chain, which occurred at 250–400 °C. Wt. loss is 22.02 and 20.01% in terms of WPS-rGO-CuO:2% and WPS-rGO-CuO:15%, however, WPS shows 73.36% wt. loss at 250–400 °C. The residual masses of WPS, WPS-rGO-CuO:2% and WPS-rGO-CuO:15% are 0.32, 10.03 and 25.04 at 600 °C, respectively. The results show that all the composite samples are thermally more stable than WPS.

### 3.4. Electrical Conductance

The incorporation of nano-sized conducting fillers into insulating polymers such as polyvinyl chloride can upgrade its conduction behavior were investigated. Non conducting polymers can be converted into conducting polymers by the addition of conductive fillers. This upgradation of composite conducting properties depends on the concentration and dissipation of the fillers into the polymer matrix [58]. In order to construct a conducting composite, a conductive network was generated in the polymer matrix to attain the percolation threshold of the fillers which was used in reference to electrical conductance.

**Figure 8:**
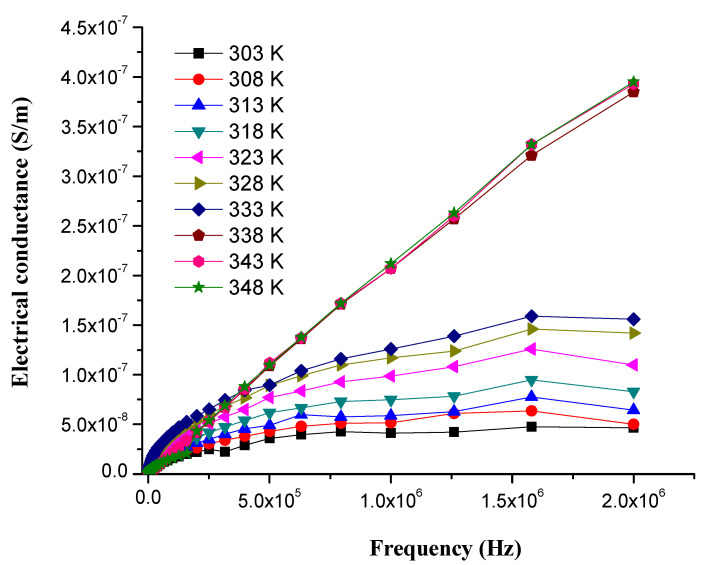
Electrical conductance of WPS-rGO-CuO: 15% composite at different temperatures.

PS is a non-conducting polymer, owing to its low electrical conductivity of 6.7 × 10^−14^ S/m. The electrical conductivity of binary composites of WPS with rGO and CuO-NPs was found at 135 S/m and 5 × 10^−11^ S/m, respectively [39]. Figure 8 shows the change in the electrical conductivity of the composite with the addition of CuO and rGO as fillers into PS matrix at frequency range of 0–2.0 × 10^6^ Hz and temperature range of 303–348 K. GO sheets provide percolated pathways for electron transfer, making the composite electrically conductive [59], similar benefits can be achieved by adding CuO-NPs. However, graphene enables the insulator to conductor transition at significantly lower loading. Results display that the electrical conductivity of WPS composites is increased by increasing the concentration of rGO and CuO-NPs as a filler [60].

**Figure 9:**
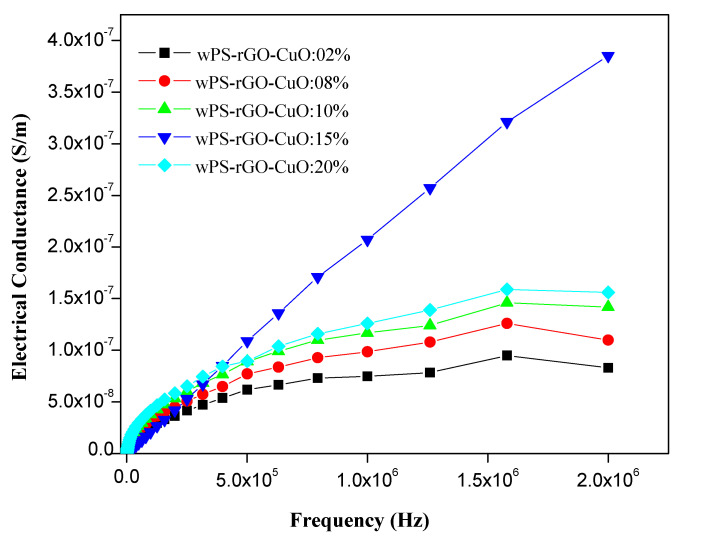
AC electrical conductance of WPS-rGO-CuO composites at varying frequency.

The percolation curve plotted against different concentrations of rGO and CuO-NPs into WPS which show an increase of electrical conductivity at different frequencies is presented in Figure 9. The electrical conductivity of WPS-rGO-CuO: 2% increased from 6.7 × 10^−14^ to 5.8 × 10^−8^ S/m. Similarly, in the cases of WPS-rGO-CuO: 8% and WPS-rGO-CuO: 10%, the electrical conductivity is increased by up to 1.0 × 10^−7^ and 1.4 × 10^−7^ S/m, respectively. The highest electrical conductivity of WPS-rGO-CuO: 15% (4.0 × 10^−7^ S/m) was observed due to the semiconducting nature of CuO-NPs and excellent electrical conductance of rGO. The mechanism of conduction shows that the fillers construct the interconnected networks of conducting paths throughout the conducting polymer matrix [61]. Nevertheless, upon further addition of the respective fillers, there is a drastic decrease in the value of electrical conductivity which is due to the agglomeration of the filler contents. In the present work, the improved electrical conduction in our materials could be used to prevent damage caused by electromagnetic rays [62].

A comparison of the electrical conductance of some polymer composites reported in literature and the current study are presented in Table 5, which reveals the WPS-rGO-CuO: 15% composite developed in the current study from a waste plastic. It shows superior electrical conductance to the reported materials.

## 4. Conclusions

In this study, WPS was used as a matrix to prepare composite with different wt. ratios of CuO-NPs and rGO as fillers. The composites were characterized by SEM, EDX, FTIR and XRD analysis. Remarkable mechanical properties were found to be enhanced with increasing the wt. ratio of rGO and CuO as fillers in WPS matrix. Composites with 15 wt.% of fillers revealed superior mechanical properties, however, further increasing of filler concentrations resulted in adverse effects. The tensile strength of the WPS-rGO-CuO: 15% composite was found with an increase of 7.655 MPa from 0.4944 MPa in the case of WPS. Similarly, the Young modulus for WPS-rGO-CuO: 15% was also increased to 1951.0 MPa from 720.37 MPa in the case of WPS. The results of electrical conductance of the binary composite of rGO and CuO-NPs showed that the nature of WPS was altered from insulator to semiconducting material upon addition up to 15% loading. Further addition led to a decrease in the S/m values. The highest electrical conductance was confirmed by WPS-rGO-CuO: 15% composite with S/m value of 4 × 10^−7^ S/m in contrast to WPS having 6.7 × 10^−14^ S/m at 2.0 × 10^6^ Hz. TGA results showed that the synthesized composites are thermally more stable than WPS with residual mass of 10.03 and 25.04% at 600 °C in contrast with WPS having residual mass of 0.32% at 600 °C.

## Figures and Tables

**Table 1:** Elemental composition of WPS, CuO-NPs, rGO and WPS-rGO-CuO composite.

Samples	Wt.% Composition					
	Experimental Wt (%)			Theoretical Wt (%)		
	C	O	Cu	C	O	Cu
WPS	94.30	5.70	-	92.25	7.74	-
CuO	-	17.34	82.64	-	16.95	80.65
rGO	91.37	8.63	-	95.05	5.5	-
WPS-rGO-CuO: 15%	89.49	5.06	5.44	89.49	5.04	5.44

**Table 2:** Mechanical properties of WPS-rGO-CuO composites with different concentrations of fillers.

Parameter	WPS	Composition of rGO-CuO (%)				
		2	8	10	15	20
Elong@Break (mm)	3.14	3.23	1.24	1.91	1.16	1.18
Elong@Yield (mm)	0.36	0.75	0.43	0.35	0.28	0.24
Strain@Break (%)	15.67	21.54	9.22	6.54	5.80	5.91
Strain@Yield (%)	2.79	5.02	2.15	1.73	1.38	1.18
Plastic Strain@Break	15.62	21.48	9.14	6.35	5.41	5.62
Load@Break (N)	0.89	0.78	1.63	5.16	31.78	1.19
Load@Yield (N)	15.61	63.28	23.83	27.93	34.27	5.08
Stress@Break(N/mm^2^)	0.49	0.54	0.64	2.87	7.66	0.66
Stress@Yield(N/mm^2^)	8.67	19.04	12.87	15.52	25.28	2.26
Youngs Modulus (N/mm^2^)	720.37	733.00	841.25	1526.5	1951.0	227.08

**Table 3:** Comparison of mechanical properties of WPS-rGO-CuO: 15% composites with reported literature.

Polymer Matrix	Fillers	Tensile Strength (N/mm^2^)	Elongation at Break (mm)	Young Modulus (N/mm^2^)	Reference
PS	Sisal fiber	0%: 34.90 20%: 25.98	0%: 9 10%: 7	0%: 380 10%: 516.83	[51]
Recycled PS	SCF and WF	0%: 1 20%: 12	0%: 7 20%: 71	0%: 24 20%: 71	[53]
Polyvinyl chloride	CaCO_3_	0%: 2825 25%: 2960	0%: 365 5%: 380	0%: 1485 20%: 1670	[54]
WPS	rGO, CuO	0%: 0.4944 15%: 7.6556	0%: 3.1380 2%: 3.2380	0%: 720.37 15%: 1951.0	Current Study

**Table 4:** Wt. loss in WPS and its composite samples at different temperature.

Sample	%wt. Loss at 50–250 °C	%wt. Loss at 250–400 °C	Residual Mass at 600 °C (g)
WPS	8.67	73.36	0.32
WPS-rGO-CuO:2%	3.52	22.02	10.03
WPS-rGO-CuO:15%	6.07	20.01	25.04

**Table 5:** Comparison of electrical conductivity of WPS-rGO-CuO: 15% composites with reported literature.

Polymer Composite	Fillers Used	Electrical Conductivity (S/m)	Reference
PVA-Al_2_O_3_: 5%	Al_2_O_3_	6.52 × 10^−5^ S/m	[60]
PMMA-Fe_2_O_3_: 2%	Fe_2_O_3_	1.55 × 10^−5^ S/m	[63]
PMMA-rGO: 2%	rGO	1.69 × 10^−10^ S/m	[49]
WPS-rGO: 2%	rGO	135 S/m	[13]
WPS-rGO-CuO: 15%	rGO/CuO	4.0 × 10^−7^ S/m	Current study

## Data Availability

All the data has been presented in this paper.
